# Supplementary material for: Resolving the full spectrum of human genome variation using Linked-Reads
Source: Genome Res. 2019 Apr;29(4):635–45. doi: 10.1101/gr.234443.118 (PMC6442396; doi:10.1101/gr.234443.118)
Supplement: Supplemental Material [file supp_gr.234443.118_Supplemental_longranger-2.2.zip › longranger-2.2.tar.bz2!/lr2.2-public/lib/stan/cmdstan/stan/doc/api/html/classstan_1_1common_1_1var__context__factory.html]

Stan: stan::common::var\_context\_factory< VARCON > Class Template Reference


|  |  |
| --- | --- |
|  | Stan  2.5.0  probability, sampling & optimization |


- Main Page
- Related Pages
- Namespaces
- Classes
- Files

- Class List
- Class Index
- Class Hierarchy
- Class Members

All Classes Namespaces Files Functions Variables Typedefs Enumerations Enumerator Friends Macros Pages

- stan
- common
- var\_context\_factory

Public Types |
Public Member Functions |
List of all members

stan::common::var\_context\_factory< VARCON > Class Template Referenceabstract

`#include <context_factory.hpp>`

|  |  |
| --- | --- |
| Public Types | |
| typedef VARCON | var\_context\_t |
|  | |

|  |  |
| --- | --- |
| Public Member Functions | |
|  | var\_context\_factory () |
|  | |
| virtual VARCON | operator() (const std::string source)=0 |
|  | |

## Detailed Description

### template<typename VARCON> class stan::common::var\_context\_factory< VARCON >

Definition at line 11 of file context\_factory.hpp.

## Member Typedef Documentation

template<typename VARCON>

typedef VARCON stan::common::var\_context\_factory< VARCON >::var\_context\_t

Definition at line 15 of file context\_factory.hpp.

## Constructor & Destructor Documentation

template<typename VARCON>

|  |  |  |  |  |
| --- | --- | --- | --- | --- |
| stan::common::var\_context\_factory< VARCON >::var\_context\_factory | ( |  | ) |  |

inline

Definition at line 13 of file context\_factory.hpp.

## Member Function Documentation

template<typename VARCON>

|  |  |  |  |  |  |
| --- | --- | --- | --- | --- | --- |
| virtual VARCON stan::common::var\_context\_factory< VARCON >::operator() | ( | const std::string | *source* | ) |  |

pure virtual

Implemented in stan::common::dump\_factory.

---

The documentation for this class was generated from the following file:

- src/stan/common/context\_factory.hpp


---

[ Stan Home Page ]

*© 2011–2014,
Stan Development Team.*
